# Can we improve outcomes in AF patients by early therapy?

**DOI:** 10.1186/1741-7015-7-72

**Published:** 2009-11-26

**Authors:** Paulus Kirchhof

**Affiliations:** 1 Department of Cardiology and Angiology, University Hospital Münster, Germany; 2 German Atrial Fibrillation competence NETwork (AFNET)

## Abstract

Atrial fibrillation affects at least 1% of the population and causes marked society-wide morbidity and mortality. Current management of atrial fibrillation including antithrombotic therapy and management of concomitant conditions in all patients, rate control therapy in most patients, and rhythm control therapy in patients with severe atrial fibrillation-related symptoms can alleviate atrial fibrillation-related symptoms but can neither effectively prevent recurrent atrial fibrillation nor suppress atrial fibrillation-related complications. Hence, there is a need for better therapy of atrial fibrillation.

The etiology of atrial fibrillation is complex. Most of the causes of atrial fibrillation which are known at present perpetuate themselves in vicious circles, and presence of the arrhythmia by itself causes marked damage of atrial myocardium. These pathophysiological insights suggest that early diagnosis and comprehensive therapy of atrial fibrillation, including adequate therapy of all atrial fibrillation-causing conditions, rate control, and rhythm control therapy, could help to prevent progression of atrial fibrillation and reduce atrial fibrillation-related complications. Such a therapy should make use of safe and effective therapeutic modalities, some of which have become available recently or will become available in the near future. The hypothesis that early diagnosis and early, comprehensive therapy of atrial fibrillation can improve outcomes requires formal testing in controlled trials.

## Commentary

**Figure 1:**
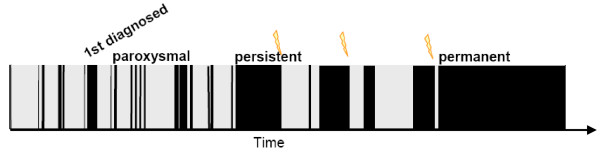
***Natural *time course of atrial fibrillation**. Shown is a typical chaotic pattern of time in atrial fibrillation (black) and time in sinus rhythm (grey) over time (x-axis). Atrial fibrillation progresses from undiagnosed to first diagnosed, paroxysmal, persistent, to permanent. Flashes indicate cardioversions as examples for therapeutic interventions that influence the *natural *time course of the arrhythmia. Reproduced with permission from [13].

Atrial fibrillation (AF) is the most common sustained arrhythmia and affects at least 1% of the population, amounting to 5.5 to 7 million patients with AF in Europe [1,2]. AF is characterized by very rapid, irregular electrical activation of the atria (350 to 400 bpm), resulting in 1) loss of coordinated contraction and transport function in the atria and 2) irregular ventricular rate and loss of ventricular rate adaptation to increased demands (arrhythmia absoluta). In an ageing population, the prevalence and incidence of AF will increase dramatically in the next decades [2,3]. Unlike most other supraventricular tachyarrhythmias, atrial fibrillation can usually not be cured [4,5]. Rather, most patients progress from paroxysmal AF, that is, AF that alternates with periods of sinus rhythm, to sustained forms (Figure 1), even when currently available therapeutic options are applied. This sobering finding is aggravated by the dramatic consequences that AF implies in affected individuals and for society (Table 1).

**Table 1:** Consequences of AF in affected individuals.

Death	death rates are doubled in AF patients. This effect is independent of known other cardiovascular risk factors or concomitant disease. Death rates are also increased in patients with a myocardial infarction or in heart failure patients when AF is added to their disease pattern.
Stroke	Approximately every 4^th ^stroke is due to AF. The possibility of "silent", undiagnosed AF - which is common in trials using systematic ECG monitoring -, may suggest that AF is also a potential cause of "cryptogenic" stroke.

Quality of life	is markedly reduced in AF patients, due to their symptoms but possibly also due to an unrecognized effect of AF on social functioning, cerebral function, or other factors. It is conceivable that AF-related hospitalizations contribute to reduced quality of life in AF patients.

Rhythm	AF causes arrhythmia absoluta and impairs rate adaptation of heart beat to demand. Abnormal heart rate can cause symptoms ranging from palpitations to acute chest pain or cardiac decompensation, especially when ventricular rate is inadequate.

Left ventricular function	is impaired by AF, especially in patients with known heart failure or with a tendency to develop heart failure. Restoring sinus rhythm and maintaining effective heart rate control can prevent such AF-induced decline of heart failure.

Other relevant outcomes in AF are indicated in [6,13].

**Figure 2:**
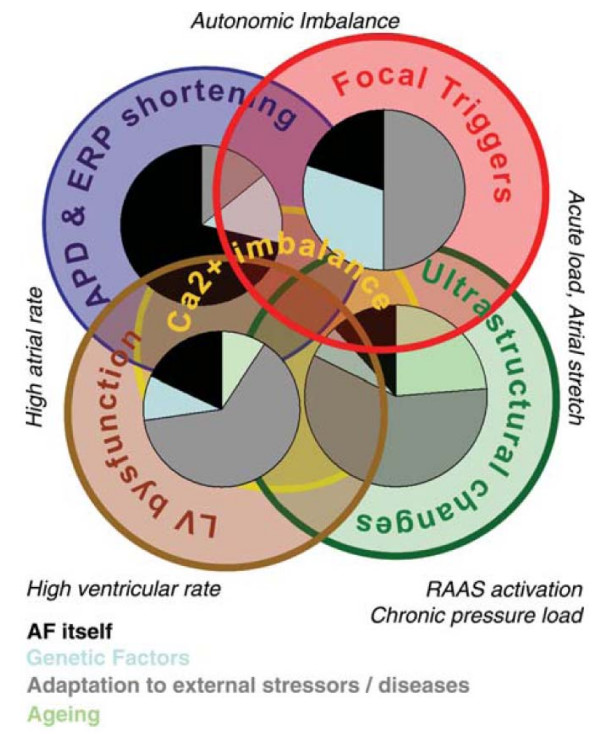
**Interdependence of four of the main mechanisms that contribute to the initiation and maintenance of atrial fibrillation**. Each circle represents a relevant factor that may initiate or perpetuate atrial fibrillation (AF): The blue circle represents shortening of the atrial action potential and effective refractory period (*electrical remodelling*), the red circle focal triggers of AF, the blue circle ultrastructural changes conferred by AF (*structural remodelling*), and the brown circle the bidirectional damage conferred by left ventricular and (left) atrial function during AF. The pie chart within each circle gives educated guesses as to how often this pathophysiological mechanism will be due to AF itself (black pie piece), genetic predispositions (light blue), a response of the atria to stressors such as hypertension, diabetes, or valvular heart disease (grey), and ageing (light green). In an individual patient (but also in a specific experimental model), AF will be due to a *blend *of these different factors as indicated by the blended overlap between the circles. Reproduced with permission from [6].

One of the reasons why AF is so difficult to cure is probably the complex etiology of the arrhythmia as recently outlined in two consensus conferences on AF [6,7]: AF can be caused by atrial structural damage conferred by cardiac or extracardiac disease and rapid irregular ventricular rate [8,9], a sign of *natural ageing *of the heart, and an *electrical accident *due to abnormal electrical function of the heart. Furthermore, AF perpetuates itself by altering electrical function of the atria (Figure 2). Unfortunately, all of these mechanisms form vicious circles and are severely aggravated once AF has manifested. Usually, several of these factors have to concur before the arrhythmia develops.

### Current management of AF patients

Adequate therapy of patients with AF considers stroke risk, often requiring continuous oral anticoagulation [4,10-12], AF-related symptoms as, for example, estimated by the European Heart Rhythm Association (EHRA) score [13], ventricular rate during AF, and the risk for AF-related complications [4]. In addition, all conditions that can contribute to AF and to AF-related complications require adequate management [14]. On top of these therapeutic interventions, rhythm control therapy should be considered when AF-related symptoms are severe. The decision for rhythm control is based on individual preferences of the patient and often also considers the perceived risk of AF recurrences [4,5,15-17].

### Potential benefits of sinus rhythm

Potentially, sinus rhythm should be able to prevent the adverse outcomes of AF: In cohort studies, AF associates with death and stroke in the population [1], and AF associates with worse prognosis than sinus rhythm in the setting of an acute myocardial infarction [18] or in patients with heart failure [19-21]. The recently published ATHENA trial also supports this notion by showing that dronedarone, an antiarrhythmic drug with additional rate-slowing properties, prevents a composite outcome of cardiovascular hospitalizations and death [22].

These observations have spurred several controlled trials testing whether rhythm control therapy improves outcomes in AF patients when compared to rate control therapy: At least six controlled trials have been completed and published [17,23-27]. None of these trials showed an advantage of rhythm control over rate control, and a meta analysis even suggested a slight benefit of rate control therapy [28].

### Why does rhythm control therapy not translate into better outcomes?

Several issues warrant consideration in the struggle to explain this unexpected finding.

1. The therapies applied in the published trials were not effective enough to really maintain sinus rhythm: In the large Atrial Fibrillation Follow-up Investigation of Rhythm Management (AFFIRM) trial, sinus rhythm rate was 60% in the rhythm control group at the end of follow-up, and 30% in the rate control group [24]. In the more recent Atrial Fibrillation and Congestive Heart Failure (AF-CHF) trial, sinus rhythm rates were around 60% in the rhythm control group and 25% in the rate control group during follow-up [17].

2. Antiarrhythmic drugs, especially when used as long-term therapy, carry a risk of proarrhythmia.

3. Apparent presence of sinus rhythm may have triggered withdrawal of prognostic therapy, for example, antithrombotic therapy, despite the fact that AF may recur silently and cause severe complications.

4. Antiarrhythmic drugs only target one of the mechanisms that cause AF (blue circle in Figure 2). Catheter ablation of AF mainly eliminates focal triggers of AF (red circle in Figure 2). A combination therapy antiarrhythmic drugs and AF ablation procedures has not been tested, let alone a *comprehensive *therapy including management of other cardiac disease.

5. Many patients were enrolled in the published trials at an advanced stage of AF, often with a long AF history.

### Can early therapy of AF help to improve efficacy and safety of rhythm control therapy?

Atrial fibrillation sustains itself through a complex process that is initiated by high atrial rate, cytosolic calcium overload, metabolic depletion, contractile dysfunction, and counter-regulatory processes that attempt to maintain function of the myocardial cells [6]. When AF is maintained for short periods of time only (minutes to hours), these processes can be reversed by restoring sinus rhythm. After several days or even weeks of the arrhythmia, irreversible atrial damage already develops. Early restoration and maintenance of sinus rhythm could prevent such irreversible AF-induced atrial damage.

Clinical observations support this concept: Conversion of AF to sinus rhythm by antiarrhythmic drugs is relatively effective when AF duration is short [29,30], but almost never effective when AF duration exceeds two weeks [4]. On a similar line, catheter ablation has a higher success rate in patients with paroxysmal AF compared to patients with sustained forms of the arrhythmia [31]. Hence, rhythm control therapy may become more effective when it is initiated early [32].

### Can earlier diagnosis of AF help to prevent AF-related complications?

It is very likely that, in addition to the patients with known AF, a large number of additional patients suffer from undiagnosed, *silent *AF [13]. An active search for AF in patients at risk for the arrhythmia may allow diagnosis of *silent *AF before the first complication occurs [33,34]. While conventional Holter electrocardiogram (ECG) recordings have a low diagnostic yield for paroxysmal AF, newer technologies like patient-operated or telemetric ECG systems, long-term Holter monitors, or even implanted ECG monitors carry the promise of allowing an early diagnosis of silent AF [6,35-37]. Early diagnosis of AF would not only allow the early initiation of rate and rhythm control therapy, but could also help to prevent AF-related complications, for example, by timely initiation of antithrombotic therapy.

### Can rhythm control therapy be delivered safely?

Early therapy of AF, especially therapy aiming at preventing complications rather than alleviating symptoms, needs to be reasonably safe, and actually safer than therapy that is demanded by a suffering patient. Safety has, however, been a major consideration in antiarrhythmic drug therapy [38]. One example is the AFFIRM trial population in which presence of sinus rhythm during follow-up associated with better survival, while therapy with antiarrhythmic drugs associated with earlier death [39]. One potential way to resolve this difficulty is to limit antiarrhythmic drug therapies to periods when their effects are really needed [40-42]. Another perspective may be to develop new, safer antiarrhythmic agents [22]. At present, the safety profile of catheter ablation of AF in relation to its efficacy has not been clearly delineated: Success rates are high, especially in paroxysmal AF patients, but complication rates are not negligible [43-45], and although most complications can be managed without long-term sequelae, there is a residual death rate induced by AF ablation [45].

## Summary

Although atrial fibrillation is clearly associated with severe consequences such as stroke, heart failure, and death, current rhythm management in AF patients does not translate into improved outcomes. Earlier initiation of rhythm control therapy, especially when embedded in a comprehensive AF management strategy, has the potential to maintain sinus rhythm more effectively, to disrupt the vicious circles that maintain AF and cause its complications, and to prevent AF-related complications before they occur. Potentially, a combination of intermittent, short-term antiarrhythmic drug therapy, antiarrhythmic drug therapy using new, potentially safer agents, and catheter ablation of AF could be components of early rhythm control therapy. Given the potential benefits and risk of such a therapy, there is a clear need to test whether early rhythm control therapy of AF can improve outcomes in AF patients.

## Competing interests

I have received consulting fees or honoraria from 3 M Medica, ASTRAZENECA, Bayer Healthcare, Boehringer Ingelheim, MEDA Pharma, Medtronic, SANOFI-Aventis, Servier, Siemens, TAKEDA. I have received research grants from Medtronic, OMRON, St Jude Medical, the German Federal Ministry for Education and Research (BMBF), Fondation LeDucq, and the German Resarch Foundation (DFG). I am or have been principle Principal Investigator of the following trials: APAL; MOBIPAPA; Flec-SL. I am member of the steering committee of the BMBF-funded AFNET and member of the board of EHRA.

## Pre-publication history

The pre-publication history for this paper can be accessed here:

http://www.biomedcentral.com/1741-7015/7/72/prepub
